# Changes in patient-sharing patterns after oncologist departures in rural and urban settings: a Medicare cohort study

**DOI:** 10.1007/s41109-025-00762-3

**Published:** 2025-12-02

**Authors:** Sarah L. Cornelius, A. James O’Malley, Gabriel A. Brooks, Anna N. A. Tosteson, Andrew Schaefer, Erika L. Moen

**Affiliations:** 1 Department of Biomedical Data Science, Geisel School of Medicine at Dartmouth, 1 Medical Center Drive, Lebanon, NH 03756 USA; 2 The Dartmouth Institute for Health Policy and Clinical Practice, Geisel School of Medicine at Dartmouth, Lebanon, NH USA; 3 Dartmouth Cancer Center, Lebanon, NH USA; 4 Department of Medicine, Geisel School of Medicine at Dartmouth, Lebanon, NH USA

**Keywords:** Physician patient-sharing networks, Oncology, Rural health

## Abstract

Cancer care relies on effective coordination within a multidisciplinary care team. Changes to teams due to departures remain understudied despite rising oncologist turnover in the United States. In this study, we aimed to investigate the impact of oncologist departures on the remaining care team members. We used Medicare claims associated with beneficiaries aged 66–99 to identify physicians involved in care for common cancer types (i.e., breast, lung, and colorectal cancer). We restricted our analysis to medical oncologists, radiation oncologists, and surgeons specializing in oncology (collectively, “oncologists”). We identified oncologists who left a practice location in 2017–2019 using the Medicare Carrier file and linked them to retained oncologists based on shared patients. Multivariable hierarchical linear regression was used to investigate how retained oncologists’ patient-sharing patterns changed after a colleague’s departure. Our results support that retained rural-practicing oncologists experienced an expansion and restructuring of their patient-sharing ties following oncologist departures while retained urban-practicing oncologists experienced a consolidation. Network restructuring may demonstrate an adaptive response that ensures patient continuity of care, but it may also reflect unique challenges faced by oncologists practicing in rural versus urban settings.

**Supplementary Information:**

The online version contains supplementary material available at 10.1007/s41109-025-00762-3.

## Introduction

Care teams, multiteam systems, and teaming are evidence-based pillars of effective cancer care (Mathieu 2023). According to the National Academies of Science, Engineering, and Medicine, teams are a vital component of high-quality cancer care across the cancer care continuum. As treatments have evolved with advancements in precision medicine, cancer care teams have grown in complexity and often require coordination among multidisciplinary specialists (Selby et al. 2019). Effective teamwork contributes to improved care coordination (Rosen et al. 2018), team climate, and team performance (Orrantia et al. 2022). As a result, barriers to effective teams can have a widespread impact on both patients and clinicians.

The growing need for coordinated cancer care is occurring within a U.S. health care system that is experiencing climbing physician turnover rates (Bond et al. 2023). Workforce turnover, which encompasses the changes in the workforce when a clinician departs from or joins an institution, is inextricably linked to care team effectiveness. While recruiting a new clinician to a care team can build new collaborations and spread ideas, departures can be expensive, disrupt referral networks, and worsen staffing shortages (Misra-Hebert et al. 2004; Atkinson et al. 2006; Pappas et al. 2022; Miller et al. 2019). The economic, safety, and patient satisfaction concerns related to physician departures are well-established in other fields (Atkinson et al. 2006; Gill and Mainous 1998; Shin et al. 2014; Shanafelt et al. 2017; Hysong et al. 2019), but there are few studies within oncology. Addressing this gap in the literature is urgently needed in light of the increasing rates of oncologists reporting intentions to leave practice, retire early, and reduce work hours following the COVID-19 pandemic (Schenkel et al. 2023, 2025).

Patient-sharing network analysis is one method for studying multidisciplinary physician teams in oncology. These networks connect physicians based on having clinical encounters with common patients. Measures can be calculated from these networks to estimate a physician’s positional importance and highlight areas of network vulnerability (Bae et al. 2015; DuGoff et al. 2018a; Barnett et al. 2011; Moen and Bynum 2019). Physician patient-sharing networks are positively correlated with physician self-report of professional relationships (Barnett et al. 2011) and patient-reported experiences of care coordination (Moen and Bynum 2019).

In this study, we aimed to estimate the extent to which oncologist departures impact the structure of the care team by analyzing changes in physician patient-sharing networks. We created annual networks using Medicare fee-for-service claims from 2016 to 2019 and calculated four measures of physician connectedness: node strength, local transitivity, Burt’s constraint, and linchpin score. Node strength, which is the sum of patient-sharing weighted ties with other physicians, estimates the strength of connections between physicians (Hevey 2018; Barrat et al. 2004). In a sub-analysis, we also calculated node strength separately for established, or persistent, patient-sharing relationships and for new relationships. Local transitivity measures the extent to which a physician’s connections are also connected to each other, forming a closed triadic structure (Watts and Strogatz 1998; Bhattacharya et al. 2023). High transitivity is hypothesized to support care coordination and greater interconnectedness in the team (Moen and Bynum 2019). Similarly, Burt’s constraint measures the extent to which a physician’s connections are interconnected (Burt 2004; Everett and Borgatti 2020). A high value indicates that a physician is in a tightly-knit, or closed, network while lower values suggest they are in a less interconnected, or open, network. The linchpin score assesses how connected an oncologist’s peers are to other oncologists of the same clinical specialty as the focal oncologist (Nemesure et al. 2021). For example, the linchpin score of a medical oncologist assesses the extent to which the physicians they share patients with are connected to another medical oncologist. Linchpin oncologists indicate local scarcity of their specialty, and thus, are considered to increase network vulnerability to a departure (Moen et al. 2022). We chose these measures as we hypothesized that physician departures would disrupt referral pathways among the remaining care team members (node strength and Burt’s constraint), decrease care coordination (local transitivity), and increase specialist scarcity (linchpin score).

As rural health care systems are already vulnerable to departures due to having a more limited oncology workforce (Levit et al. 2020; Shulman et al. 2020), we hypothesized that retained oncologists practicing in rural settings would experience greater burden following a colleague’s departure compared with their more urban counterparts. Understanding the impacts of oncologist departures on those who remain is an important first step to building and supporting a robust oncology workforce.

## Methods

### Data source and patient cohort

**Fig. 1:**
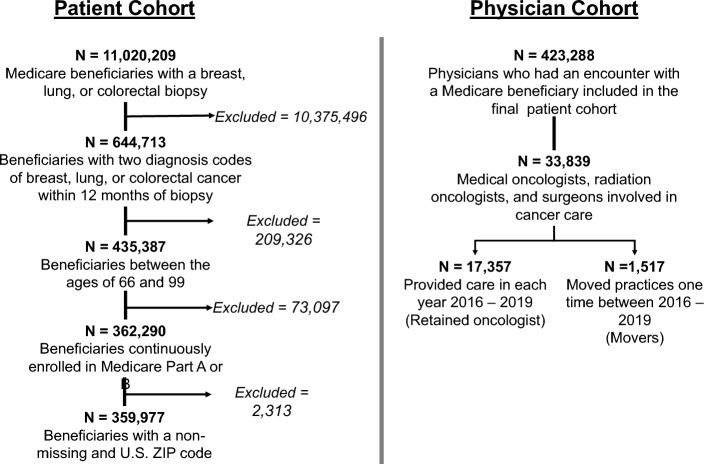
CONSORT diagram. We identified 11,020,209 Medicare beneficiaries with a biopsy of breast, lung, or colorectal cancer in 2016–2019. Following restrictions, we developed a cohort of medical oncologists, radiation oncologists, and surgeons involved in cancer care

Our study included 100% fee-for-service Medicare data from 2016 to 2019. We first developed a patient cohort, which included Medicare fee-for-service beneficiaries diagnosed with incident breast (International Classification of Diseases [ICD]-10: C50.x), lung (ICD-10: C34.x), or colorectal (ICD-10: C18.x, C19, C20) cancer between 2016 and 2019 (Fig. 1). We chose to focus on breast, lung, and colorectal cancers because these cancers are among the most common cancer types in the U.S. (National Cancer Institute 2025). Consistent with prior studies, we required patients to have a diagnostic biopsy followed by two cancer diagnosis codes on separate days within 12 months. (Bronson et al. 2018) Prevalent cases were excluded using a 12-month look-back period. Patients were also required to be age 66–99 years at the time of biopsy and continuously enrolled in Parts A and B for the 12 months following and 12 months prior to their biopsy.

### Physician cohort

We then built a physician cohort based on all physicians who cared for these patients between the 3 months prior to and 12 months following their diagnostic biopsy procedure, as identified from encounters in the Medicare Provider Analysis and Review (MedPAR) and Carrier files. The MedPAR file contains information on all inpatient stays covered by Medicare Part A, and the Carrier file contains information on any service covered by Medicare Part B provided in an office, hospital, or other medical site.

Physicians specializing in oncology were identified using taxonomy codes listed from the National Plan and Provider Enumeration System (NPPES), a file that provides information about all organizations and providers with a National Provider Identifier (NPI). The first three taxonomy codes were used to identify medical oncologists, radiation oncologists, and surgeons, who will collectively be referred to as “oncologists” in this paper (Cornelius et al. 2024). Surgeons were also required to have performed a cancer-directed surgical procedure on a patient in our cohort to confirm specialization in oncology. Our analytic (i.e., physician) cohort was restricted to oncologists who were present throughout all four years of the study to enrich for retained oncologists. Departing oncologists, described below, were considered retained in the years prior to their departure.

### Identifying oncologist departures

We identified oncologists who moved between practice locations based on changes in their billing location (Cornelius et al. 2025). We sorted each oncologist’s Carrier claims by date and identified whether the billing ZIP code changed from the prior encounter. We summed the number of times an oncologist’s location changed during each calendar year. Oncologists were considered to have moved if there was only one change during the calendar year, supporting the assumption that the oncologist practiced consistently in one location before transitioning entirely to a second location. Some oncologists regularly practice in multiple locations, such as oncologists who provide rural outreach; (Scodari et al. 2024) thus, these oncologists have multiple transitions between ZIP codes during a calendar year. To capture oncologists who may have practiced in more than one location either before or after a more distant move, we linked the billing ZIP code of each encounter to its associated hospital referral region (HRR). Because HRRs represent regional health care markets or systems, they often include several hospitals or health care clinics. Once linked, we summed the number of times an oncologist’s HRR changed over a calendar year and considered oncologists to have moved if they changed their HRR one time during the year.

Departures were then derived based on the ZIP code from which the oncologist moved. Oncologists with departures in multiple years were excluded due to the potential instability of their measures over the study timeframe.

### Physician patient-sharing networks

We built an undirected nationwide physician patient-sharing network for each year of the study. Physicians of all specialties who had encounters with patients in our cohort were included in the network and connected to other physicians based on shared patients during the calendar year. The edge weights in the network were counts of the number of shared patients for each physician pair during the calendar year and were included in the calculations for node strength and linchpin score.

### Outcome measures

Our primary outcome measures were short-term changes in network (i.e., node strength, local transitivity, Burt’s constraint, and linchpin score; Supplemental Table 1) and clinical characteristics (i.e., annual cohort patient volume). These changes were calculated as the difference in the characteristic between the year of and the year prior to the departure.

*Node strength:* Measures the extent to which an oncologist shared patients with other physicians in the network, and was used to estimate connectedness (Hevey 2018; Barrat et al. 2004; Moen et al. 2016) As it is the sum of edge weights, values can range from 0 to infinity.

*Local transitivity:* Measures the proportion of an oncologist’s connections that also share patients with each other (i.e., to create a closed triadic patient-sharing relationship), and has been used to estimate care coordination (Hevey 2018; Watts and Strogatz 1998; Bhattacharya et al. 2023; Hollingsworth et al. 2016).

*Burt’s constraint:* Measures the extent of interconnectedness among a physician’s direct connections, with higher values indicating the physician is more closed off, or constrained, in their direct network (Burt 2004; Everett and Borgatti 2020).

*Linchpin score:* Measures the extent to which a physician is locally unique for their specialty.(Nemesure et al. 2021) For example, a medical oncologists with a high linchpin score have peers who lack ties to other medical oncologists.

*Patient volume:* The number of unique cohort patients with whom the oncologist had encounters.

In secondary analyses, we calculated node strength separately for established, or persistent, ties and for new ties in order to further assess how oncologists changed their patient-sharing patterns after a colleague’s departure. For each calendar year, we identified persistent ties as physicians who shared at least one patient in the year of and the year prior to the observation year (DuGoff et al. 2018b). New ties were physician pairs that did not exist in the prior year. Node strength was then calculated for both subsets of connections by summing the edge weights for all persistent or new ties to the focal physician.

### Exposure variables

For each oncologist, we counted the number of departing oncologists to whom they were connected (please see Supplemental Fig. 1 for a flow diagram outlining exposure and outcome variable classification). Oncologists who shared at least two patients with a departing oncologist during the year of and the year prior to the departure were considered to have a direct connection. Prior work has shown that requiring at least two shared patients optimizes reliability and inclusivity of physician peer groups (Herrin et al. 2019). In sensitivity analyses, we also required thresholds of three, four, and five shared patients. As we required this one-year look-back, we restricted our analytic cohort to physicians who billed for services in 2017–2019. Secondary exposures included counts of two specific types of departing oncologist connections that we hypothesized would have a greater impact on the retained oncologists: (1) departing oncologists of the same clinical specialty (i.e., medical oncology, radiation oncology, or surgery) as the retained oncologist; and (2) departing oncologists who were more unique for their specialty within their local networks (i.e., linchpin oncologists). An oncologist was considered a linchpin oncologist if their linchpin score was in the top 15% for their specialty (Moen et al. 2022).

### Covariates

We obtained each oncologist’s sex and specialty from the NPPES file. Physician rurality was based on the Rural Urban Commuting Area (RUCA) codes linked to the ZIP code associated with the plurality of their care for retained oncologists and the ZIP code where the oncologist departed from for departing oncologists. Rurality was dichotomized based on the University of Washington’s Categorization C methodology, which outlines urban versus non-urban status based on population and commuting patterns (WWAMI Rural Health Research Center. RUCA Data: Using RUCA Data xxxx). We calculated the racial and ethnic composition of each physician’s patient panel using the Medicare Beneficiary Enrollment file, which includes demographic and plan information on Medicare beneficiaries. This was calculated as the percent of patients who were Hispanic, non-Hispanic black, and non-Hispanic white.

### Statistical analysis

We calculated standardized mean differences (SMD) to identify baseline (i.e., 2016) differences among our physician groups. The groups were stratified by whether the oncologist was ever or never connected to a departing oncologist in 2017–2019. We calculated propensity scores with multivariable logistic regression in which the dependent variable was an indicator for whether the oncologist was ever or never connected to a departing oncologist and the independent variables included all covariates with a univariate SMD of 0.1 or greater. We used inverse-probability of treatment weighting (IPTW) to balance our observation groups in the estimation of the statistical models described in the following paragraphs.

Our primary analysis used multivariable hierarchical linear regression to compare changes in network and practice characteristics by connection status to departing oncologists. All models were estimated using the IPTW weights as design weights, which reflected the propensity for a physician to be connected to a departing oncologist. We conducted separate regression models for each of the primary outcome measures. Each model included random intercepts for HRR to account for geographic clustering and for NPI to account for repeated measurements of oncologists over the three observation years. Due to conducting multiple regression models, we used a Bonferroni correction when determining statistical significance. To obtain a family-wise type I error rate for a two-sided test of 5%, this reduced our significance threshold for individual tests to *p* < 0.01. These models were then repeated for each secondary exposure (i.e., counts of connections to departing oncologists of the same specialty and departing linchpin oncologists).

An exploratory analysis was completed to investigate pre- and post-departure changes in node strength among persistent and new ties. As we required a one-year lookback to identify persistent versus new ties, we limited to oncologists who were connected to at least one departing oncologist in 2018 or 2019. We conducted a paired t-test incorporating our IPTW estimated weights to determine whether there were significant changes in these node strength measures prior to and following a colleague’s departure while inflating our standard errors to reflect the reduction of effective sample-size in a weighted sampled versus an unweighted sample.

All statistical analyses were conducted using RStudio version 4.3.1. This study was determined to be exempt by Dartmouth College Committee for the Protection of Human Subjects.

## Results

We included 359,977 Medicare beneficiaries who met all entrance criteria for our patient cohort (Fig. 1). The majority of patients were female (78.7%), were diagnosed with breast cancer (56.3%), and were non-Hispanic white race (87.0%; Supplemental Table 2). This patient cohort informed our physician cohort, which included 7150 medical oncologists, 3229 radiation oncologists, and 8495 surgeons. Among this total of 18,874 oncologists in our cohort, 2023 (10.7%) were connected to at least one departing oncologist between 2017 and 2019 (Table 1). Physician sex (SMD, 0.193) and specialty (SMD, 0.638) had a moderate effect on being connected to departing oncologists. Baseline patient volume (SMD, 0.813), node strength (SMD, 0.642), local transitivity (SMD, 0.612), Burt’s constraint (SMD, 0.514), and linchpin score (SMD, 0.269) showed moderate to large effects of being connected versus never being connected to a departing oncologist. These covariates also had a modest effect on being a departed (versus retained) oncologist (Supplemental Table 3).

**Table 1:** Baseline characteristics of oncologists who were ever versus never connected to oncologist movers between 2017 and 2019

Characteristic^a^	Never connected (N = 16,851)	Ever connected (N = 2,023)	Unweighted SMD	Weighted SMD^b^
*Demographic characteristics*				
Physician male sex	12,313 (73.1%)	1,298 (64.2%)	0.193	0.059
*Specialty*				
Medical oncologist	6,266 (37.2%)	884 (43.7%)	0.638	0.088
Radiation oncologist	2,524 (15.0%)	705 (34.8%)		
Surgeon	8,061 (47.8%)	434 (21.5%)		
Rural practice location	1,907 (11.3%)	167 (8.3%)	0.103	0.025
*Composition of patient panel*				
Hispanic, mean (SD)	0.9% (6.19%)	0.7% (3.5%)	0.047	0.017
Non-Hispanic black, mean (SD)	6.9% (16.6%)	6.8% (12.8%)	0.005	0.040
Non-Hispanic white, mean (SD)	85.9% (22.2%)	86.7% (16.5%)	0.041	0.054
*Baseline practice and network characteristics*				
Patient volume				
Low (< 5)	7,349 (43.6%)	279 (13.8%)	0.813	0.081
Medium (5–9)	4,897 (29.1%)	516 (25.5%)		
High (≥ 10)	4,605 (27.3%)	1,228 (60.7%)		
Node strength, mean (SD)	81.2 (90.9)	153.0 (130.0)	0.642	0.053
Local transitivity, mean (SD)	0.536 (0.226)	0.418 (0.150)	0.612	0.097
Burt’s constraint, mean (SD)	0.100 (0.091)	0.064 (0.036)	0.514	0.145
Linchpin score, mean (SD)	0.134 (0.135)	0.101 (0.108)	0.269	0.040

^a^Characteristics summarized as number (%) unless otherwise specified; ^b^Weighted using inverse probability treatment weighting (IPTW) with propensity score of Ever Being Connected estimated using physician sex, specialty, patient volume, node strength, local transitivity, Burt’s constraint, and linchpin score as predictors

SD, standard deviation; SMD, standardized mean difference

For each additional connection to a departing oncologist, rural oncologists’ node strength increased by an average of 7.8 (99% CI, 3.1–12.6; *p* < 0.001) and patient volume increased by 1.3 patients (99% CI, 0.7–1.8; *p* < 0.001; Table 2). These changes in node strength and patient volume were more pronounced when the retained and departing oncologists shared the same specialty. For each connection to departing oncologists of the same specialty, rural oncologists’ node strength increased by 32.3 (99% CI, 19.1–45.6; *p* < 0.001) and patient volume increased by 5.1 patients (99% CI, 3.6–6.5; *p* < 0.001; Table 3). Retained oncologists also experienced a decrease of 0.066 in their local transitivity (99% CI, − 0.100 to − 0.031; *p* < 0.001) after a colleague of the same specialty departed. Similarly, the impact of movement was more pronounced as the threshold for connections was increased (Supplemental Table 4). Retained rural oncologists also experienced small, yet significant changes after the departure of a linchpin colleague. For each connection to a departing linchpin oncologist, retained oncologists experienced a decrease of 0.050 (99% CI, − 0.091 to − 0.008; *p* = 0.002) in local transitivity and an increase of 2.2 (99% CI, 0.5–3.9; *p* = 0.001) in patient volume (Supplemental Table 5).

**Table 2:** Changes in retained oncologists’ patient-sharing network and practice characteristics for each connection to any mover

	Rural	Urban		
	Pre/post-departure change^a,b^ (99% CI)	*p* value	Pre/post-departure change^a,b^ (99% CI)	*p* value
Node strength	**7.8 (3.1, 12.6)**	< 0.001	− 1.7 (− 4.1, 0.7)	0.065
Local transitivity	− 0.002 (− 0.015, 0.010)	0.649	**0.017 (0.013, 0.021)**	< 0.001
Burt’s constraint	− 0.0002 (− 0.007, 0.006)	0.927	**0.003 (0.002, 0.005)**	< 0.001
Linchpin score	− 0.008 (− 0.018, 0.002)	0.042	**0.005 (0.002, 0.007)**	< 0.001
Patient volume	**1.3 (0.7, 1.8)**	< 0.001	− 0.04 (− 0.3, 0.2)	0.675

^a^Weighted using inverse probability treatment weighting (IPTW) with the propensity score of Ever Being Connected estimated using physician sex, specialty, patient volume, and 2016 observations for node strength, local transitivity, Burt’s constraint, and linchpin score as predictors; ^b^**Bold** values are statistically significant at *p* < .01

**Table 3:** Changes in retained oncologists’ patient-sharing network and practice characteristics for each connection to a mover of the same specialty

	Rural	Urban		
	Pre/post-departure change^a,b^ (99% CI)	*p* value	Pre/post-departure change^a,b^ (99% CI)	*p* value
Node strength	**32.3 (19.1, 45.6)**	< 0.001	3.6 (− 1.4, 8.7)	0.060
Local transitivity	**− 0.066 (− 0.100, − 0.031)**	< 0.001	**0.013 (0.004, 0.021)**	< 0.001
Burt’s constraint	− 0.003 (− 0.021, 0.015)	0.660	0.002 (− 0.002, 0.006)	0.178
Linchpin score	0.015 (− 0.013, 0.043)	0.175	**0.008 (0.003, 0.013)**	< 0.001
Patient volume	**5.1 (3.6, 6.5)**	< 0.001	**0.5 (0.1, 1.0)**	< 0.001

^a^Weighted using inverse probability treatment weighting (IPTW) with the propensity score of Ever Being Connected estimated using physician sex, specialty, patient volume, and 2016 observations for node strength, local transitivity, Burt’s constraint, and linchpin score as predictors; ^b^**Bold** values are statistically significant at *p* < .01

Retained urban-practicing oncologists experienced small but statistically significant increases in their local transitivity (0.017; 99% CI, 0.013–0.021; *p* < 0.001), Burt’s constraint (0.003; 99% CI, 0.002–0.005; *p* < 0.001), and linchpin score (0.005; 99% CI, 0.003–0.007; *p* < 0.001) with each additional departing oncologist connection (Table 2). When the departing and retained oncologists shared the same specialty, the retained oncologist experienced increases in local transitivity (0.013; 99% CI, 0.004–0.021; *p* < 0.001), linchpin score (0.008; 99% CI, 0.003–0.013; *p* < 0.001), and patient volume (0.5; 99% CI, 0.1–1.0; *p* < 0.001) following their colleague’s departure (Table 3). These results were similar with increasing thresholds for connections (Supplemental Table 4). With each connection to a departing linchpin oncologist, retained urban-practicing oncologists experienced a decrease of 22.9 (99% CI, − 34.2 to − 11.52; *p* < 0.001) in node strength and 2.1 (99% CI, − 3.2 to − 1.1; *p* < 0.001) in patient volume as well as an increase of 0.040 (99% CI, 0.021–0.059; *p* < 0.001) in local transitivity (Supplemental Table 5).

Considering the consistent changes in retained oncologists’ node strength following oncologist departures in rural and urban settings, we further investigated whether these changes reflected persistent relationships (more patient-sharing with established care team members) or new patient-sharing relationships (creating relationships with new care team members). Node strength with persistent ties increased for both rural- (mean change, 16.4; standard deviation [SD], 40.0; *p* < 0.001) and urban-practicing (mean change, 27.1; SD, 66.9; *p* < 0.001) retained oncologists, while only retained rural-practicing oncologists experienced an increase in node strength with new ties (mean change, 2.3; SD, 35.7; *p* = 0.017) following a colleague’s departure (Table 4).

**Table 4:** Node strength changes prior to and after a departure among retained oncologists who were connected to at least one departing oncologist in 2018–2019

	Rural				Urban			
	Pre-departure	Post-departure	Change	*p* value	Pre-departure	Post-departure	Change	*p* value
Node strength with persistent ties, mean (SD)	69.6 (80.3)	85.9 (96.9)	16.4 (40.0)	< 0.001	108.6 (125.0)	135.7 (147.6)	27.1 (66.9)	< 0.001
Node strength with new ties, mean (SD)	59.0 (39.1)	61.3 (42.4)	2.3 (35.7)	0.017	100.8 (72.1)	101.5 (70.8)	0.6 (56.2)	0.241

^a^Weighted using inverse probability treatment weighting (IPTW) with the propensity score of Ever Being Connected estimated using physician sex, specialty, patient volume, and 2016 observations for node strength, local transitivity, Burt’s constraint, and linchpin score as predictors

SD, standard deviation

## Discussion

In this retrospective longitudinal cohort study, we used Medicare fee-for-service claims to examine the impact of oncologist departures on the structure of the remaining care team in rural and urban settings. We found that oncologists practicing in rural settings experienced increases in patient volume and patient-sharing following the departure of an oncology colleague. Perhaps most strikingly, when a rural-practicing oncologist was of the same specialty as the departing physician, their study cohort patient volume increased by 5 patients and their overall sum of shared patients across all ties increased by 32 for each departure. Conversely, physicians practicing in urban settings experienced increased clustering (i.e., local transitivity), linchpin score, and constraint, with results being consistent when the departing and retained oncologists shared the same specialty. This study identified important differences in the impact of oncologist departures between rural and urban care teams.

**Fig. 2:**
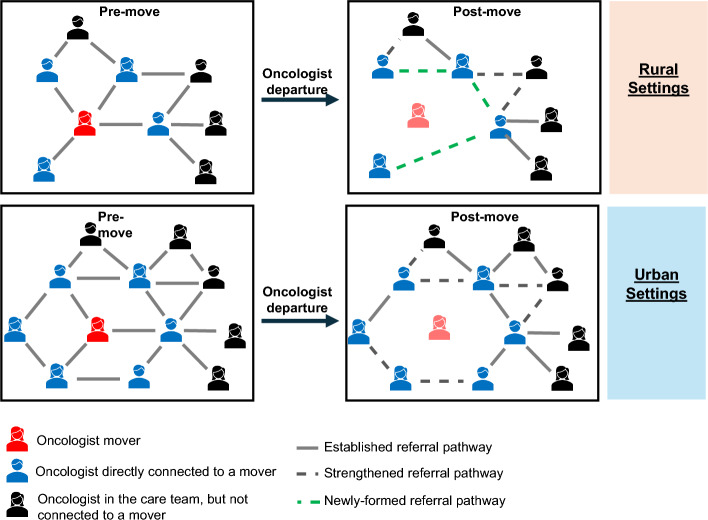
Hypothesized effects of departures on physician patient-sharing networks. Each example network contains a departing oncologist (red), and oncologists who are directly (blue) and indirectly (black) connected to them. Edges are based on shared patients. Established connections (gray) were present prior to the oncologist’s departure while strengthened (dashed gray) and new connections (dashed green) formed after the departure. Rural cancer care teams are often sparser, resulting in the new referral pathways being formed after an oncologist’s departure. Urban cancer care teams are often larger and contain more redundancy within their workforce, allowing the care team to continue largely unchanged after an oncologist’s departure

We posit that the increase in patient volume and patient-sharing experienced by oncologists practicing in rural settings may contribute to increased clinical burden on the remaining care team members. Following a departure, rural-practicing physicians take on new patients, build new patient-sharing ties, and strengthen their existing ties in order to maintain their ability to care for their patients. While increased node strength is typically associated with increased connectedness (Moen et al. 2016; Landon et al. 2012; Forstner et al. 2023), in the case of departures, it may also be signaling a restructuring within the care team (see hypothesized network change, Fig. 2). For each additional departure, retained oncologists’ professional networks continued to expand. This demonstrates resilience within the care team if it indicates a transition that ensured patients continued to receive their necessary treatment. However, restructuring may create vulnerabilities in health care systems with repeated departures. As turnover continues, there is an increased burden to build new referral pathways to ensure patient care is not impacted. Furthermore, while these oncologists may take on more patients in response to a colleague’s departure, they may also receive new referrals from physicians who hadn’t previously referred patients to them. For example, some patients may be seen by different providers due to changes in treatment planning that would not have occurred in a stable oncologist network (Franko and Frankova 2020).

These results bolster the growing evidence that rural oncology teams respond to physician departures in a unique way, and thus, there may be specific interventions that can support rural practices (Moen et al. 2025). For example, it is well established that physicians require system- and regional-level resources to deliver care effectively (Mathieu 2023; Noyes et al. 2016). As this study highlighted how rural oncologists build new patient-sharing ties after a colleague’s departure, rural care teams may especially benefit when there are established collaborative relationships with teams at local facilities or community clinics. These established connections may reduce a physician’s clinical burden by allowing them to more easily identify and collaborate with physicians with whom they did not previously collaborate. Additionally, patients may be able to be triaged more easily, allowing more complex cases to remain at the referral center while less complex cases could be referred to providers in the community.

Rural health care systems are inherently vulnerable to oncologist departures as they are often more spread out, have fewer generalists and specialists, and fewer cancer-specific resources, such as dedicated cancer centers (Levit et al. 2020; Skinner et al. 2019). These rural settings often manage a broader spectrum of oncological services while urban settings are better equipped to treat more complex cases requiring more specialized care (Bhatia et al. 2022). Furthermore, while nearly 25% of the U.S. population live in a rural setting, only 10% of oncologists practice in one, resulting in substantial geographic variation in access to cancer specialists across the U.S (Snapshot: State of the Oncology Workforce in America 2025; Ramakrishnan and Suandi 2024). Considering these access issues, it is critical to further study the widespread impacts of oncologist turnover, particularly within in rural settings.

In contrast, retained urban-practicing oncologists experienced increased local transitivity, constraint, and linchpin score (i.e., specialist scarcity) following a colleague’s departure. This supports that rather than an increase in patient-sharing, urban oncologists’ professional networks became slightly more consolidated with each oncologist departure. With larger care teams, there is often more redundancy within the workforce, which allows care teams to continue functioning despite the departure (Nemesure et al. 2021; Moen et al. 2022; Aboagye et al. 2014). This could allow for referral pathways to continue with little restructuring until a new oncologist can be recruited to the open position. However, even among care teams with a fairly robust response to departures, they may experience vulnerabilities when a unique specialist leaves. After the departure of a linchpin oncologist, retained urban-practicing oncologists experienced increases in local transitivity, supporting this consolidation in their ties, in addition to substantial decreases in their patient volume and node strength. Linchpin physicians introduce vulnerability within a health system due to the lower redundancy of their specialty in the network. With reductions in patient volume and patient-sharing, it is possible that the short-term loss of a specialist led to reduced access to cancer care for some patients, supporting findings from prior work (Franko and Frankova 2020). Future studies should investigate whether patients experience reduced access to cancer treatments, even if only temporarily, after the departure of a linchpin oncologist.

The results of this study are directly applicable to care teams treating common cancer types (i.e., breast, colorectal, and lung cancer). However, several studies beyond cancer have reported that turnover, or related “personnel flux,” creates disruptions for the staff above and beyond short staffing (Miller et al. 2019; Summers et al. 2012). Changes to the structure of care teams likely contribute to these disruptions, particularly in rural settings. Considering the established impact of “personnel flux” in other fields, we believe these results are generalizable to oncology more broadly. Furthermore, these results may generalize to other health care settings, particularly those requiring multidisciplinary teams.

### Limitations

While a strength of our study was the use of claims data to identify departures and patient-sharing patterns, these data are also subject to important limitations. First, our cohort of oncologists was based on those who billed for services for patients with breast, colorectal, or lung cancer and may not fully generalize to other cancer types. We expect that patient-sharing networks for rare cancers would change more substantially following departures due to more regionalized care from fewer subspecialist physicians. Second, our cohort included departures resulting from movement. As a result, we may be underestimating the number and effect of departing oncologists on our cohort. There are many situations that lead to a departure, such as retirement, but movement is a substantial concern that is potentially modifiable. Third, our classification method to identify movement relied on billing location rather than facility location. It is possible that some movers were misclassified as non-movers, particularly if they practiced in multiple locations. We aimed to limit this misclassification by also defining movement status based on transitions between HRRs. This allowed physicians to transition frequently between ZIP codes within the same HRR while still capturing movement status of more distant moves (i.e., a transition from one HRR to another). Fourth, rural and urban settings differ in many ways that are not captured in Medicare claims. For example, facilities in both settings can have different resources available to clinicians, including resources that improve collaboration between clinicians. Future work may consider focusing on hospitals within one health care system to better account for this potential for residual confounding. Fifth, we did not prioritize connections to movers based on the proportional strength of their connection, although this would bring important nuance to future studies focused on the impact of movers. Sixth, we created undirected patient-sharing networks rather than directed networks for this study. There is currently no established method for defining directed patient-sharing networks using claims data as there is an infinite way of doing so. As we did not have a clear hypothesis for how directionality of the network measures would be impacted by a departure, we chose to use undirected networks for this analysis. Recent studies have provided guidance for constructing directed patient-sharing networks in a more optimal way and so may inform future work that accounts for directionality (O’Malley et al. 2023, 2025). Finally, our data precedes the COVID-19 pandemic and so our study does not capture the changes in the oncology workforce during or after the pandemic.

## Conclusions

Our results revealed significant changes in patient sharing and patient volume among retained rural- and urban-practicing oncologists after the departure of an oncologist colleague. While rural oncologists experienced an expansion and restructuring of their professional ties, urban oncologists experienced a consolidation of their ties. Strategies to support a robust oncology workforce will benefit from considering the role of practice rurality when anticipating and addressing changes experienced by care teams due to departures.

## Supplementary Information

Additional file1 (R 4 KB)

Additional file2 (R 3 KB)

Additional file3 (R 5 KB)

Additional file4 (DOCX 219 KB)

Additional file5 (PPTX 51 KB)

## Data Availability

The data that support the findings of this study are available from the Centers for Medicare and Medicaid Services (CMS). Access to these data require a data use agreement between the research team and the Research Data Assistance Center (ResDAC) and so are not publicly available. The authors will provide code upon reasonable request and with permission of ResDAC.

## Abbreviations

CMS: Centers for Medicare and Medicaid Services
HRR: Hospital Referral Region
ICD: International Classification of Diseases
IPTW: Inverse-probability of treatment weighting
MedPAR: Medicare Provider Analysis and Review
NPPES: National Plan and Provider Enumeration System
ResDAC: Research Data Assistance Center
RUCA: Rural urban commuting area
SMD: Standardized mean difference
