# Supplementary material for: Changes in patient-sharing patterns after oncologist departures in rural and urban settings: a Medicare cohort study
Source: Appl Netw Sci. 2025 Dec 2;11(1):1. doi: 10.1007/s41109-025-00762-3 (PMC12775101; doi:10.1007/s41109-025-00762-3)
Supplement: Supplementary file 4 — Additional file4 (DOCX 219 KB) [file 41109_2025_762_MOESM4_ESM.docx]

**Supplemental Materials**

Supplemental Figure

| 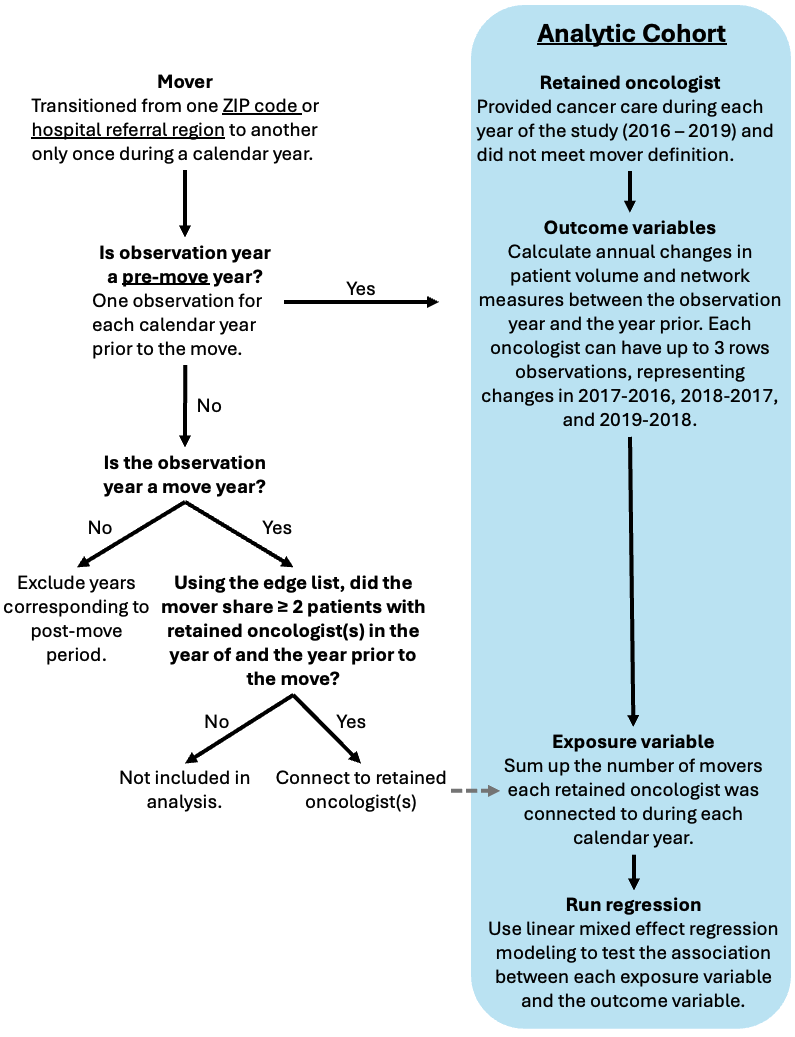 |
| --- |
| **Supplemental Figure 1. Outline of study methods.** We built a cohort of medical oncologists, radiation oncologists, and surgeons, and we classified them as retained during the study period or as a mover. If the oncologist was a mover, we considered them retained for all years during the pre-move period. We excluded all years during the post-move period. For movers during the pre-move years and retained oncologists, we calculated the change in patient volume and networks characteristics for each calendar year of the study period. During the move year, we linked each mover to retained oncologists if they shared at least two patients in the year of and the year prior to the move. We summed the number of movers with whom each retained oncologist was connected and conducted linear mixed effect regression for each exposure variable. |

Supplemental Tables:

| Supplemental Table 1. The definition and purpose of outcome measures used. | | |  |
| --- | --- | --- | --- |
| Network Measure | Definition | Purpose | Range |
| Node strength | The sum of the edge weights, or number of shared patients, each oncology physician has with other physicians in the network. | To measure the connectedness of physicians. | 0 to infinity |
| Local transitivity | The proportion of clusters of three physicians (i.e., triads) that a physician is in that are closed. | To measure the degree of clustering within a physician’s direct ties, which can estimate the capacity for coordinated care. | 0 to 1 |
| Burt’s constraint | The proportion of a node's neighbors that are connected to non-redundant (unconnected) others. | To measure the extent to which a physician is in a closed (i.e., constrained) or open network. | 0 to 1 |
| Linchpin score | The proportion of a physician’s peers that are connected to other physicians of the same specialty as the focal physician. | To measure redundancy or scarcity of oncology physicians in a physician’s care team, which can estimate vulnerability within the workforce. | 0 to 1 |

| Supplemental Table 2. Demographic characteristics of patients included in patient cohort. | |
| --- | --- |
| Characteristic^a^ | Overall  (N=359,977)^b^ |
| Cancer cohort |  |
| Breast | 202,700 (56.3%) |
| Colorectal | 82,906 (23.0%) |
| Lung | 69,516 (19.3%) |
| Sex |  |
| Female | 283,227 (78.7%) |
| Male | 71,895 (20.0%) |
| Race/Ethnicity |  |
| Hispanic | 2,565 (0.7%) |
| Non-Hispanic black | 23,820 (6.6%) |
| Non-Hispanic white | 313,319 (87.0%) |
| Other | 15,418 (4.3%) |
| Age |  |
| 66-69 | 85,809 (23.8%) |
| 70-74 | 105,932 (29.4%) |
| 75-79 | 80,467 (22.4%) |
| 80+ | 82,914 (23.0%) |
| Charlson comorbidity index |  |
| 0 | 162,981 (45.3%) |
| 1 | 84,808 (23.6%) |
| 2+ | 107,303 (29.8%) |
| ^a^Characteristics summarized as number (%) unless otherwise specified; ^b^Demographic information was missing for 4,855 beneficiaries. | |

| Supplemental Table 3. Baseline 2016 characteristics of retained and departing oncology physicians between 2017 - 2019. | | | |
| --- | --- | --- | --- |
| Characteristic^a^ | Retained  (N=17,357) | Departing  (N=1,517) | Unweighted SMD |
| Demographic Characteristics | | | |
| Physician male sex | 12,603 (72.6%) | 1,008 (66.4%) | 0.134 |
| Specialty |  |  |  |
| Medical oncologist | 6,589 (38.0%) | 561 (37.0%) | 0.139 |
| Radiation oncologist | 3,031 (17.5%) | 198 (13.1%) |  |
| Surgeon | 7,737 (44.6%) | 758 (50.0%) |  |
| Rural practice location | 1,907 (11.0%) | 167 (11.0%) | 0.001 |
| Composition of patient panel |  |  |  |
| Hispanic, mean (SD) | 0.9% (5.8%) | 1.0% (7.6%) | 0.024 |
| Non-Hispanic black, mean (SD) | 6.7% (16.0%) | 8.4% (18.9%) | 0.095 |
| Non-Hispanic white, mean (SD) | 86.1% (21.4%) | 84.8% (24.0%) | 0.057 |
| Baseline Practice and Network Characteristics | | | |
| Patient volume |  |  |  |
| Low (<5) | 6,902 (39.8%) | 726 (47.9%) | 0.185 |
| Medium (5-9) | 4,990 (28.7%) | 423 (27.9%) |  |
| High (≥10) | 5,465 (31.5%) | 368 (24.3%) |  |
| Node strength, mean (SD) | 90.6 (100.0) | 69.5 (67.4) | 0.247 |
| Local transitivity, mean (SD) | 0.520 (0.221) | 0.557 (0.229) | 0.164 |
| Burt’s constraint, mean (SD) | 0.095 (0.087) | 0.105 (0.089) | 0.114 |
| Linchpin score, mean (SD) | 0.130 (0.133) | 0.139 (0.134) | 0.066 |
| ^a^Characteristics summarized as number (%) unless otherwise specified  Abbreviations: SD, standard deviation; SMD, standardized mean difference. | | | |

| Supplemental Table 4. Changes in retained oncologists’ patient-sharing network and practice characteristics for each connection to a mover based on increasingly strict requirements for a connection. | | | | |
| --- | --- | --- | --- | --- |
|  | Rural | | Urban | |
|  | Pre/post-departure change^a,b^  Estimate (99% CI) | p-value | Pre/post-departure change^a,b^  Estimate (99% CI) | p-value |
| Each connection requires *three* shared patients | | | | |
| Node strength | **12.4 (5.2, 19.6)** | <0.001 | -1.7 (-5.1, 1.6) | 0.179 |
| Local transitivity | 0.012 (-0.007, 0.031) | 0.115 | **0.014 (0.008, 0.020)** | <0.001 |
| Burt’s constraint | 0.002 (-0.008, 0.012) | 0.594 | **0.003 (0.001, 0.006)** | <0.001 |
| Linchpin score | **-0.017 (-0.032, -0.001)** | 0.005 | **0.004 (0.0004, 0.007)** | 0.004 |
| Patient volume | **1.5 (0.7, 2.3)** | <0.001 | 0.1 (-0.2, 0.4) | 0.238 |
| Each connection requires *four* shared patients | | | | |
| Node strength | **12.2 (2.2, 22.2)** | 0.002 | -2.8 (-7.2, 1.7) | 0.108 |
| Local transitivity | 0.001 (-0.025, 0.027) | 0.933 | **0.018 (0.011, 0.026)** | <0.001 |
| Burt’s constraint | -0.002 (-0.016, 0.011) | 0.661 | **0.004 (0.001, 0.007)** | 0.001 |
| Linchpin score | **-0.054 (-0.075, -0.033)** | <0.001 | **0.005 (0.00001, 0.009)** | 0.010 |
| Patient volume | **1.4 (0.3, 2.5)** | 0.001 | 0.2 (-0.2, 0.6) | 0.283 |
| Each connection requires *five* shared patients | | | | |
| Node strength | **33.5 (20.1, 46.9)** | <0.001 | -5.6 (-11.4, 0.2) | 0.013 |
| Local transitivity | 0.012 (-0.023, 0.048) | 0.378 | **0.025 (0.015, 0.034)** | <0.001 |
| Burt’s constraint | 0.0002 (-0.018, 0.018) | 0.973 | **0.005 (0.001, 0.010)** | 0.001 |
| Linchpin score | **-0.088 (-0.116, -0.060)** | <0.001 | 0.005 (-0.001, 0.011) | 0.041 |
| Patient volume | **3.8 (2.4, 5.3)** | <0.001 | 0.1 (-0.4, 0.7) | 0.495 |
| ^a^Weighted using inverse probability treatment weighting (IPTW) with the propensity score of Ever Being Connected estimated using physician sex, specialty, patient volume, and 2016 observations for node strength, local transitivity, Burt’s constraint, and linchpin score as predictors; ^b^**Bold** values are statistically significant at p < .01. | | | | |

| Supplemental Table 5. Changes in retained oncologists’ patient-sharing network and practice characteristics for each connection to a linchpin oncologist mover. | | | | |
| --- | --- | --- | --- | --- |
|  | Rural | | Urban | |
|  | Pre/post-departure change^a,b^  Estimate (99% CI) | p-value | Pre/post-departure change^a,b^  Estimate (99% CI) | p-value |
| Node strength | 3.5 (-12.3, 19.3) | 0.568 | **-22.9 (-34.2, -11.5)** | <0.001 |
| Local transitivity | **-0.050 (-0.091, -0.008)** | 0.002 | **0.040 (0.021, 0.059)** | <0.001 |
| Burt’s constraint | -0.005 (-0.026, 0.016) | 0.505 | 0.005 (-0.004, 0.013) | 0.151 |
| Linchpin score | 0.009 (-0.024, 0.042) | 0.487 | 0.008 (-0.003, 0.020) | 0.062 |
| Patient volume | **2.2 (0.5, 3.9)** | 0.001 | **-2.1 (-3.2, -1.1)** | <0.001 |
| ^a^Weighted using inverse probability treatment weighting (IPTW) with the propensity score of Ever Being Connected estimated using physician sex, specialty, patient volume, and 2016 observations for node strength, local transitivity, Burt’s constraint, and linchpin score as predictors; ^b^**Bold** values are statistically significant at p < .01. | | | | |
