# Supplementary material for: Changes in patient-sharing patterns after oncologist departures in rural and urban settings: a Medicare cohort study
Source: Appl Netw Sci. 2025 Dec 2;11(1):1. doi: 10.1007/s41109-025-00762-3 (PMC12775101; doi:10.1007/s41109-025-00762-3)
Supplement: Supplementary file 5 — Additional file5 (PPTX 51 KB) [file 41109_2025_762_MOESM5_ESM.pptx]

## Slide 1
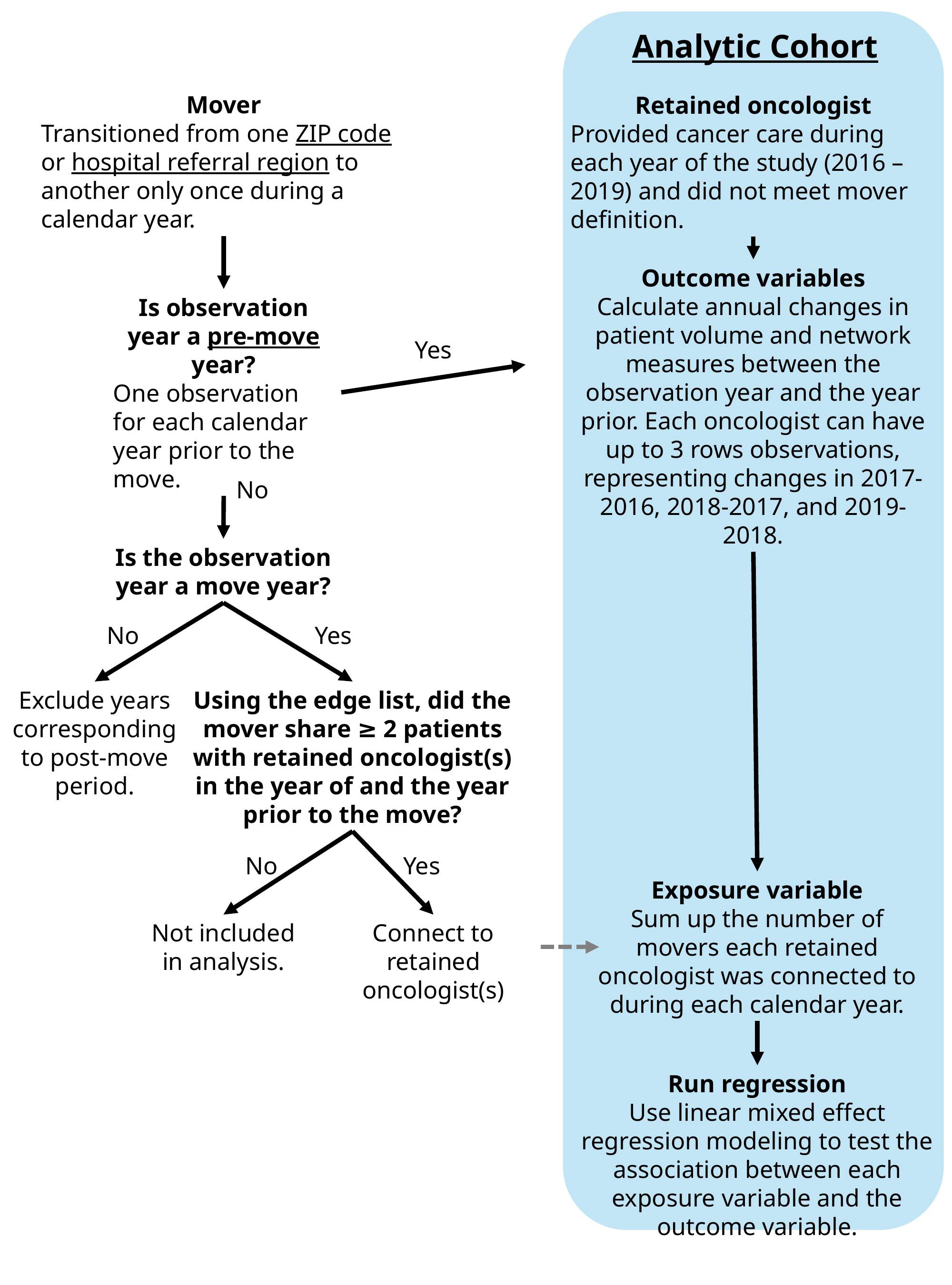

Analytic Cohort
Mover
Transitioned from one ZIP code or hospital referral region to another only once during a calendar year.
Retained oncologist
Provided cancer care during each year of the study (2016 – 2019) and did not meet mover definition.
Outcome variables
Calculate annual changes in patient volume and network measures between the observation year and the year prior. Each oncologist can have up to 3 rows observations, representing changes in 2017-2016, 2018-2017, and 2019-2018.
Is observation year a pre-move year?
One observation for each calendar year prior to the move.
Yes
No
Is the observation year a move year?
No
Yes
Exclude years corresponding to post-move period.
Using the edge list, did the mover share ≥ 2 patients with retained oncologist(s) in the year of and the year prior to the move?
Yes
No
Exposure variable
Sum up the number of movers each retained oncologist was connected to during each calendar year.
Not included in analysis.
Connect to retained oncologist(s)
Run regression
Use linear mixed effect regression modeling to test the association between each exposure variable and the outcome variable.
